# Long-term efficacy and safety of dupilumab for moderate-to-severe atopic dermatitis: a prospective real-world cohort study in China

**DOI:** 10.3389/fimmu.2024.1419164

**Published:** 2024-11-01

**Authors:** Yuyi Wang, Ruiling Jia, Qin Hu, Xiao Tao, Qi He, Guangying Luo, Qiong Xiong, Zhongyu Zhang, Yujuan Xiao, Yi Liu

**Affiliations:** ^1^ Department of Dermatology, The First Affiliated Hospital of Chongqing College of Traditional Chinese Medicine, Chongqing, China; ^2^ Department of Dermatology, Chongqing Hospital of Traditional Chinese Medicine, Chongqing, China; ^3^ Chongqing Key Laboratory of Integrative Dermatology Research, Chongqing, China; ^4^ Chongqing Clinical Research Center for Dermatology, Chongqing, China

**Keywords:** atopic dermatitis, dupilumab, long-term efficacy, safety, influencing factors, real-world study

## Abstract

**Backgrounds:**

Dupilumab has demonstrated remarkable efficacy and safety in clinical trials for moderate-to-severe atopic dermatitis (AD). However, long-term real-world evidence, especially in the Chinese population, remains limited.

**Objective:**

To investigate the long-term efficacy and safety of dupilumab for moderate-to-severe AD in a real-world clinical setting in China and analyze factors that may influence its long-term treatment outcomes.

**Methods:**

This prospective, observational real-world study included moderate-to-severe AD patients from the AD cohort of the dermatology department of Chongqing Hospital of Traditional Chinese Medicine who received dupilumab treatment for≥52 weeks. Efficacy and adverse events were assessed at baseline, weeks 4, 16, 24, and 52. Multivariate logistic regression analysis was used to identify predictive factors for achieving EASI 50 and EASI 75 at week 52.

**Results:**

A total of 124 patients were included. At week 52, EASI, SCORAD, IGA, NRS, and DLQI scores were significantly improved compared to baseline. The proportions of patients achieving EASI-50/75 were 50.81%/29.84%, 72.58%/42.74%, 75%/53.23%, and 67.74%/41.94% at weeks 4, 16, 24 and 52, respectively. Female sex, absence of atopic comorbidities, higher baseline EASI, and medication compliance were positive predictive factors for 52-week EASI-50/75. Eosinophil elevation predicted lower EASI-50 attainment. Nineteen adverse events occurred during the 52-week period (incidence rate: 14.52%), mostly mild and manageable.

**Conclusions:**

Dupilumab demonstrated significant efficacy and a low incidence of adverse events over 52 weeks in Chinese patients with moderate-to-severe AD, making it an effective and safe long-term treatment option. Predictive factors were identified to guide treatment optimization.

## 1 Introduction

Atopic dermatitis (AD) is a chronic, recurrent, inflammatory skin disease that often starts in childhood but can continue into adulthood or develop later in life (1). The condition is characterized primarily by dry skin and eczematous lesions, which can appear anywhere on the body but are most commonly found on the hands, feet, ankles, wrists, neck, upper chest, eyelids, and in the folds of the elbows and knees (2–4). Moderate-to-severe AD is often accompanied by intense pruritus, which not only leads to repeated scratching of the lesions but also results in impaired sleep quality and severely affects the patient’s quality of life (5–7). Globally, AD affects up to 20% of children and 2 to 10% of adults (1, 8). In recent years, the overall prevalence of AD has been increasing. In China, a 2016 survey found that the prevalence among outpatients was 7.8% (9), while another study targeting children aged 1 to 7 years revealed a prevalence of 12.94% (10). Notably, the incidence of AD in the elderly population has been continuously rising. However, diagnosing AD in older patients can be challenging, as it often presents differently from childhood-onset AD, lacking the typical lesions and distribution. To exclude other serious conditions such as adverse drug reactions, seborrheic dermatitis, prurigo nodularis, and cutaneous T-cell lymphoma, healthcare providers usually need to perform blood tests, histopathological examinations, and patch tests when assessing elderly patients with suspected AD (11).

The pathophysiology of AD is complex and involves skin barrier dysfunctions, immune dysregulation, and susceptibility to colonization of pathogenic microbes (1). A critical part of its mechanism appears to be due to overactivity of Th2 cells, a type of T lymphocyte that plays an integral role in the immune system response (12–14). Th2 cells, especially in the acute phase of AD, produce certain cytokines such as Interleukin-4 (IL-4), Interleukin-13 (IL-13), and Interleukin-31 (IL-31), all of which have been identified as substantial contributors to the development and aggravation of AD (15–17). IL-4 and IL-13, in particular, are involved in stimulating immunoglobulin E (IgE) synthesis, which initiates and amplifies the inflammatory process in AD (18). Dupilumab is a monoclonal antibody directed against the shared receptor alpha chain subunit (IL-4Rα) utilized by IL-4 and IL-13 to mediate downstream signaling (19). By binding to IL-4Rα, dupilumab inhibits the ability of IL-4 and IL-13 to interact with their receptor complex (20). This interferes with activation of inflammatory processes dependent on IL-4/13 signaling (20). It is this specified, targeted action on the pathological immune response that makes Dupilumab an effective treatment for AD (21). In clinical trials, dupilumab provided significant improvement in disease severity, symptoms, and quality of life for moderate-to-severe AD patients (22–25).

In 2020, the China National Medical Products Administration (CNMPA) approved dupilumab for the treatment of moderate-to-severe atopic dermatitis (AD) (26). Following its inclusion in the national medical insurance system in 2021, the number of Chinese AD patients receiving dupilumab treatment increased significantly, owing to improved accessibility and affordability provided by medical insurance coverage. Dupilumab has exhibited therapeutic efficacy and an acceptable safety profile, addressing a critical unmet need for many Chinese AD patients who are refractory to conventional treatments (26). Although preliminary real-world data from China demonstrates the short-term (16 weeks) efficacy of dupilumab in treating AD, longitudinal observations assessing long-term (52 weeks) outcomes in the Chinese real-world clinical setting are still lacking (27–29).

The objective of this study is to investigate the long-term efficacy and safety of dupilumab for the treatment of moderate-to-severe AD) in a real-world clinical setting in China. Furthermore, we aim to identify and analyze factors that may influence the long-term treatment outcomes of dupilumab in patients with moderate-to-severe AD.

## 2 Materials and methods

### 2.1 Study design

This prospective, observational study was part of the AD cohort from the dermatology department of Chongqing Hospital of Traditional Chinese Medicine (Chongqing, China), which consecutively enrolled all AD patients. The study was approved by the Ethics Review Committee of Chongqing Hospital of Traditional Chinese Medicine (ethics number: 2020-ky-50). All participants or their guardians signed informed consent forms.

### 2.2 Eligible population

The study included AD patients who met the following criteria: (1) diagnosed with AD according to the Hanifin & Rajka criteria (30); moderate-to-severe disease severity, defined as an Eczema Area and Severity Index (EASI) score ≥8 or a SCORing Atopic Dermatitis (SCORAD) score ≥25 (31); and (3) treated with dupilumab for at least 52 weeks within the cohort. Patients who discontinued dupilumab treatment for more than 8 weeks were excluded from the study. There were no age or gender restrictions for patient inclusion.

### 2.3 Dupilumab dosing regimen

For adults and children/adolescents weighing ≥ 60 kg, the initial dosage was 600 mg, followed by a subcutaneous injection of 300 mg every two weeks. For children/adolescents aged 6-17 years: those weighing 30-60 kg were given an initial dose of 400 mg, followed by 200 mg every two weeks; those weighing 15 to <30 kg were given an initial dose of 600 mg, followed by 300 mg every four weeks. For children under 6 years: those weighing 5 to <15 kg, a dosage of 200 mg every four weeks was recommended; for those weighing 15-30 kg, a dosage of 300 mg every four weeks was prescribed, given as a subcutaneous injection.

After 16 weeks of treatment, clinicians could extend the dosing interval of dupilumab to 4 weeks for patients who had achieved EASI 75 or above. Additionally, it was permissible for patients to extend the treatment interval due to personal reasons, such as work commitments (26).

### 2.4 Dupilumab medication compliance

Medication compliance in the first 16 weeks: use of dupilumab every 2 weeks; Medication compliance from week 17 to 52: use of dupilumab every 2-4 weeks; Medication compliance throughout the entire 52 weeks: use of dupilumab every 2 weeks for the first 16 weeks and every 2-4 weeks from week 17 to 52. Pediatric patients who used dupilumab every 4 weeks within the 52-week period were considered to be medication compliant.

### 2.5 Follow-up and assessment

**Figure 1:**
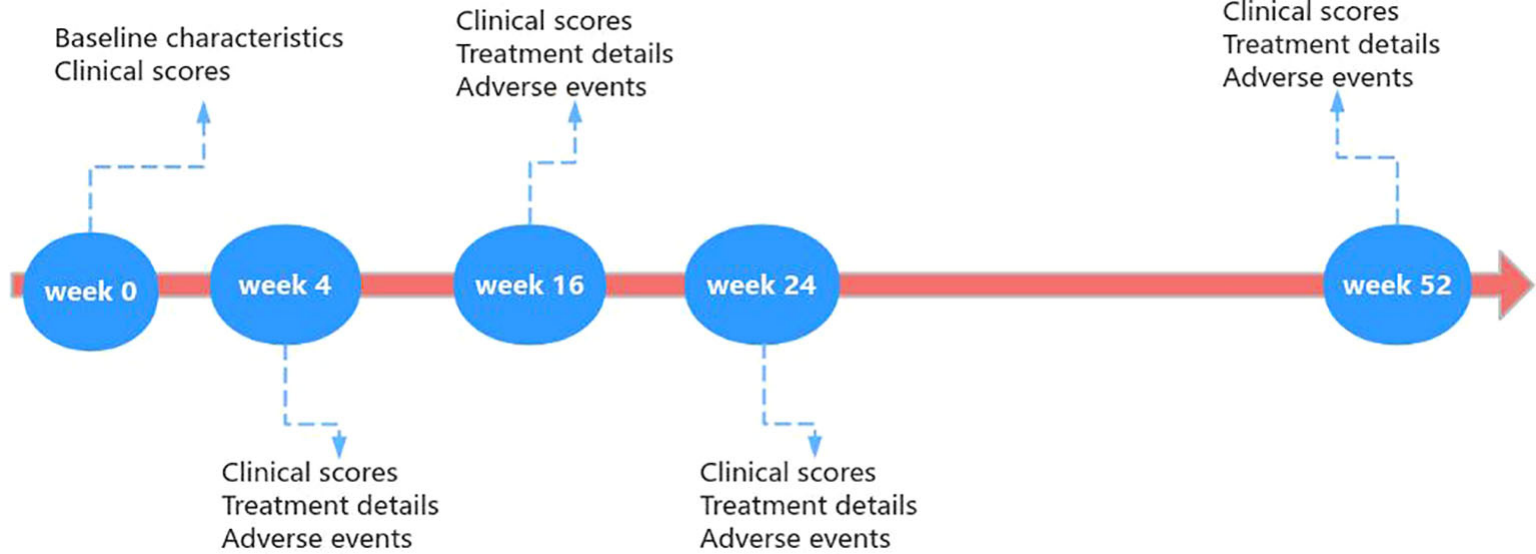
Flow chart of follow-up.

Clinicians assessed AD patients treated with dupilumab and collected data at baseline, week 4, week 16, week 24, and week 52. The specific process was illustrated in **Figure 1**.

The following demographic and clinical characteristics of patients were recorded at baseline: gender, age, body mass index (BMI), age of onset, disease duration, atopic comorbidities (allergic asthma, allergic rhinitis, and allergic conjunctivitis), history of immunosuppressant use (glucocorticoids, cyclosporine A, and methotrexate), and eosinophil counts obtained from laboratory tests.

The severity of the disease was prospectively assessed using clinical scores, including the EASI (range 0-72), SCORAD (range 0-103), Investigator Global Assessment (IGA, range 0-5), Pruritus Numeric Rating Scale (NRS, range 0-10), and Dermatology Life Quality Index (DLQI, range 0-30). EASI, SCORAD, and IGA were clinician-reported outcomes assessed by clinicians, while NRS and DLQI were patient-reported outcomes. Clinical scores were evaluated at baseline and at weeks 4, 16, 24, and 52.

Throughout the entire 52-week period, the interval of dupilumab administration for each patient and any adverse events occurring during dupilumab treatment were recorded.

### 2.6 Statistical analyses

All statistical analyses were performed using R version 4.2.0. Quantitative variables were described using mean ± standard deviation (SD) or median (interquartile range, Q1-Q3), depending on the normality of the data distribution. Categorical variables were presented as counts and percentages. The distribution of each efficacy outcome during the follow-up period was illustrated using box plots, while the proportions and cumulative proportions of achieving a 50% or greater improvement in the EASI (EASI 50) and a 75% or greater improvement in the EASI (EASI 75) at each follow-up time point were depicted using line charts. Logistic regression analysis was employed to identify factors influencing the achievement of EASI 50 and EASI 75. Statistical significance was set at a P value of less than 0.05.

## 3 Results

### 3.1 Patient characteristics

Between November 2020 and June 2023, a total of 575 patients were enrolled in the AD cohort. Among them, 124 moderate-to-severe AD patients who received dupilumab treatment for at least 52 weeks and met the inclusion criteria were included in this study (**Table 1**). The remaining 451 patients were excluded for the following reasons: 238 did not use dupilumab, 178 used dupilumab but the treatment duration was less than 52 weeks, and 35 discontinued the medication for more than 8 weeks after initiating dupilumab.

**Table 1:** Patient characteristics at baseline (n = 124).

Characteristics	Value (N = 124)
Age, years, Mean (SD)	43.99 (23.42)
Age groups, n (%)	
<18 years old	18 (14.52)
18-64 years old	73 (58.87)
≥65 years old	33 (26.61)
Sex, Male,n (%)	83 (66.94)
**BMI,** Mean (SD)	21.97 (3.70)
Overweight BMI, n (%)	37 (29.84)
Age of onset, years, Mean (SD)	34.28 (24.78)
Groups of age of onset, n (%)	
<18 years old	43 (39.09)
18-64 years old	48 (43.64)
≥65 years old	19 (17.27)
Disease duration, months,Mean (SD)	116.34 (106.80)
Disease duration groups, n (%)	
<72 months	58 (46.77)
≥72 months	66 (53.23)
Atopic comorbidities, n (%)	58 (46.77)
Allergic asthma, n (%)	17 (13.71)
Allergic rhinitis, n (%)	46 (37.10)
Allergic conjunctivitis, n (%)	3 (2.42)
History of immunosuppressant use, n (%)	44 (35.48)
Glucocorticoids, n (%)	30 (24.19)
Cyclosporine A, n (%)	13 (10.48)
Methotrexate, n (%)	19 (15.32)
**EASI,** Mean (SD)	9.44 (8.26)
**SCORAD,** Mean (SD)	43.92 (12.49)
**DLQI,** Mean (SD)	12.77 (6.03)
**IGA,** Mean (SD)	3.30 (0.54)
**NRS,** Mean (SD)	6.25 (2.03)
Eosinophil counts (10⁹/L)	
n(missing)	79(45)
Mean (SD)	0.60 (0.50)

SD, Standard Deviation; BMI, Body Mass Index; EASI, Eczema Area and Severity Index; SCORAD, SCORing Atopic Dermatitis; DLQI, Dermatology Life Quality Index; IGA, Investigator’s Global Assessment; NRS, Numeric Rating Scale.

The 124 included patients consisted of 83 males (66.94%) and had an age range of 3 to 92 years, with a mean age of 43.99 years. Based on the BMI criteria of over 24, 37 patients (29.84%) were classified as overweight. The mean age of onset for the patients was 34.28 years, with 43 (39.09%) patients under 18 years old, 48 (43.64%) between 18-64 years old, and 19 (17.27%) aged 65 or older. The disease duration was less than 72 months for 58 (46.77%) patients, while the remaining 66 (53.23%) had a duration equal to or exceeding 72 months. Regarding atopic comorbidities, 17 patients had allergic asthma, 46 had allergic rhinitis, and 3 had allergic conjunctivitis. A history of immunosuppressant use revealed that 30 patients had taken oral steroids, 13 had been prescribed cyclosporine, and 19 patients had used methotrexate. Notably, 18 patients concurrently used two or three types of immunosuppressants. At baseline, the mean EASI score was 9.44, and the mean SCORAD score was 43.92. According to the SCORAD categorization of AD severity, 86 patients were assessed as having moderate AD (25<SCORAD ≤ 50), while 38 had severe AD (SCORAD>50). The detailed baseline demographics and clinical characteristics of the patients are summarized in **Table 1**.

### 3.2 Dupilumab usage details

Among the 124 AD patients treated with dupilumab for 52 weeks, full compliance was observed in only 34 patients (27.42%). Non-compliance was noted in 90 patients, including 4 who were non-compliant within the first 16 weeks, 58 who exhibited non-compliance after the 16-week mark, and 28 who remained non-compliant throughout the entire 52-week period. Concurrent use of topical medications during dupilumab treatment was observed in 91 patients (73.39%). Regarding the concurrent topical medications, 79 patients used them in combination with corticosteroids, 86 patients used them in combination with non-steroidal medications such as tacrolimus, and 18 patients used them in combination with traditional Chinese medicine. Detailed information regarding dupilumab usage is presented in **Table 2**.

**Table 2:** Dupilumab usage details for patients over 52 weeks.

Characteristics	Value (N = 124)
Dupilumab medication compliance, n (%)	
Non-compliant in first 16 weeks	4 (3.23)
Non-compliant from week 17-52	58 (46.77)
Non-compliant throughout 52 weeks	28 (22.58)
Compliant throughout 52 weeks	34 (27.42)
Concomitant medications, n (%)	91 (73.39)
Corticosteroids, n (%)	79 (63.71)
Non-corticosteroids, n (%)	86 (69.35)
Traditional Chinese medicine, n (%)	18 (14.52)

### 3.3 Efficacy

#### 3.3.1 Clinical scores

**Figure 2:**
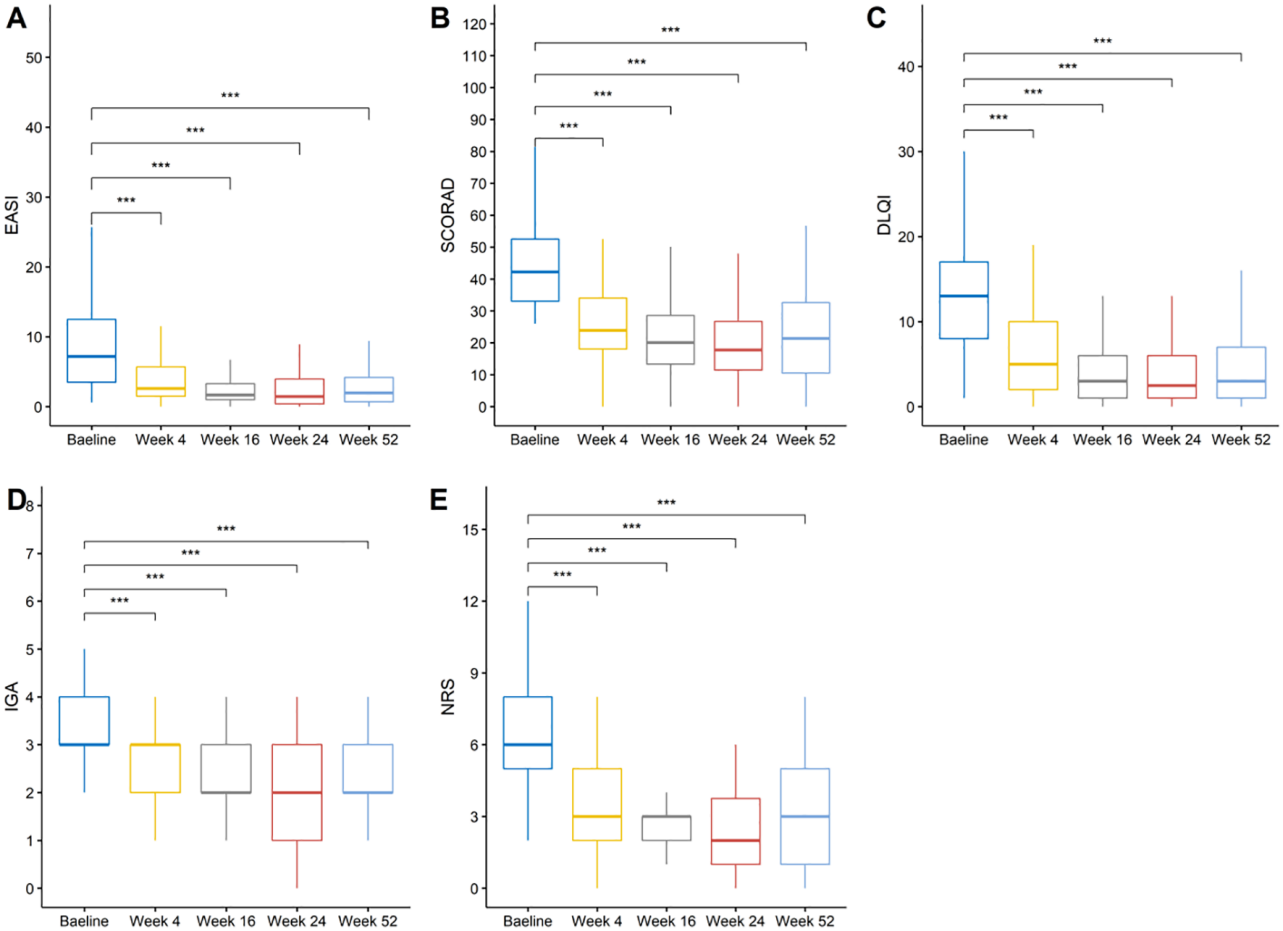
Changes in disease severity and quality of life measures over time. **(A)** EASI scores, **(B)** SCORAD index, **(C)** DLQI scores, **(D)** IGA scores, and **(E)** NRS itch scores at baseline and weeks 4, 16, 24, and 52. ***: p <= 0.001 were adjusted using the Bonferroni correction.

The clinical scores before and after treatment are summarized in **Table 3**. Dupilumab treatment resulted in rapid and sustained improvements in disease severity, symptoms, and quality of life over the 52-week treatment course. Specifically, the mean EASI score decreased from 9.44 at baseline to 4.36 (-48.88%) at week 4, 2.79 (-64.16%) at week 16, 2.51 (-67.12%) at week 24, and 2.86 (-59.66%) at week 52. Similarly, the mean SCORAD score reduced from 43.92 at baseline to 26.41 (-40.06%) at week 4, 21.16 (-51.02%) at week 16, 19.75 (-53.67%) at week 24, and 22.38 (-47.32%) at week 52. The mean IGA score declined from 3.33 at baseline to 2.48 (-24.46%) at week 4, 2.13 (-35.31%) at week 16, 1.97 (-40.42%) at week 24, and 2.08 (-35.95%) at week 52. The mean NRS score decreased from 6.25 at baseline to 3.52 (-40.55%) at week 4, 2.52 (-55.09%) at week 16, 2.54 (-55.67%) at week 24, and 3.07 (-44.91%) at week 52. The mean DLQI score reduced from 12.77 at baseline to 6.38 (-45.62%) at week 4, 4.28 (-60.16%) at week 16, 3.96 (-61.38%) at week 24, and 4.34 (-54.25%) at week 52. Notably, compared to baseline, there were statistically significant improvements in all five clinical scores at weeks 4, 16, 24, and 52 (**Figure 2**).

**Table 3:** Clinical scores before and after treatment.

Variables	Baseline	Week 4	Week 16	Week 24	Week 52	Percentage change from baseline at Week 4 (%)	Percentage change from baseline at Week 16 (%)	Percentage change from baseline at Week 24 (%)	Percentage change from baseline at Week 52(%)
EASI									
n(missing)	124(0)	124(0)	123(1)	122(2)	121(3)	124(0)	123(1)	122(2)	121(3)
Mean(SD)	9.44 (8.26)	4.36 (4.89)	2.79 (3.17)	2.51 (3.02)	2.86 (3.00)	-48.88 (40.46)	-64.16 (29.50)	-67.12 (33.04)	-59.66 (41.98)
SCORAD									
n(missing)	124(0)	124(0)	123(1)	122(2)	121(3)	124(0)	123(1)	122(2)	121(3)
Mean(SD)	43.92 (12.49)	26.41 (12.40)	21.16 (11.59)	19.75 (12.55)	22.38 (14.17)	-40.06 (22.63)	-51.02 (24.88)	-53.67 (28.95)	-47.32 (32.03)
IGA									
n(missing)	124(0)	124(0)	123(1)	122(2)	121(3)	124(0)	123(1)	122(2)	121(3)
Mean(SD)	3.33 (0.58)	2.48 (0.77)	2.13 (0.80)	1.97 (0.97)	2.08 (1.03)	-24.46 (23.72)	-35.31 (23.95)	-40.42 (29.11)	-35.95 (33.89)
NRS									
n(missing)	124(0)	124(0)	122(2)	122(2)	121(3)	124(0)	122(2)	122(2)	121(3)
Mean(SD)	6.25 (2.03)	3.52 (2.01)	2.52 (1.55)	2.54 (1.99)	3.07 (2.14)	-40.55 (35.25)	-55.09 (34.37)	-55.67 (38.26)	-44.91 (46.02)
DLQI									
n(missing)	101(23)	101(23)	88(36)	98(26)	105(19)	97(27)	80(44)	87(37)	87(37)
Mean(SD)	12.77 (6.03)	6.38 (5.02)	4.28 (3.78)	3.96 (4.03)	4.34 (4.13)	-45.62 (37.26)	-60.16 (38.23)	-61.38 (54.15)	-54.25 (59.46)

SD, Standard Deviation; EASI, Eczema Area and Severity Index; SCORAD, SCORing Atopic Dermatitis; DLQI, Dermatology Life Quality Index; IGA, Investigator’s Global Assessment; NRS, Numeric Rating Scale.

#### 3.3.2 Proportions and cumulative proportions of EASI 50 and EASI 75

**Figure 3:**
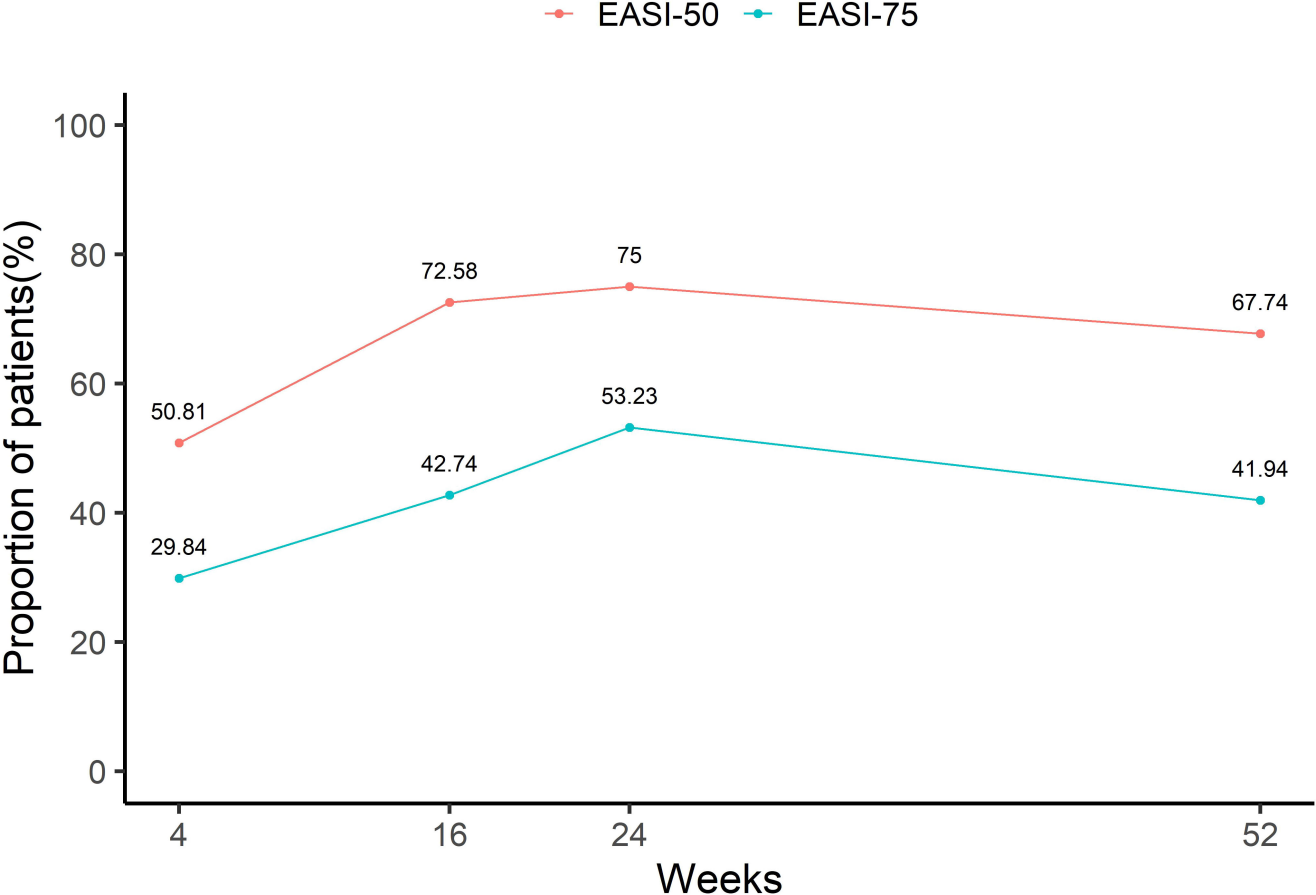
Proportions of achieving EASI50 and EASI75 at different time points.

**Figure 3** presents the proportion of patients achieving EASI 50 and EASI 75 at various time points. After 4 weeks of treatment, half of the patients (50.81%) had already achieved EASI 50, and 29.84% had achieved EASI 75. At weeks 16 and 24, the proportion of patients achieving EASI 50 and EASI 75 continued to increase, reaching 72.58% and 75% for EASI 50, and 42.74% and 53.23% for EASI 75, respectively. However, by week 52, the percentage of patients achieving EASI 50 and EASI 75 decreased slightly to 67.74% and 41.94%, respectively.

**Figure 4:**
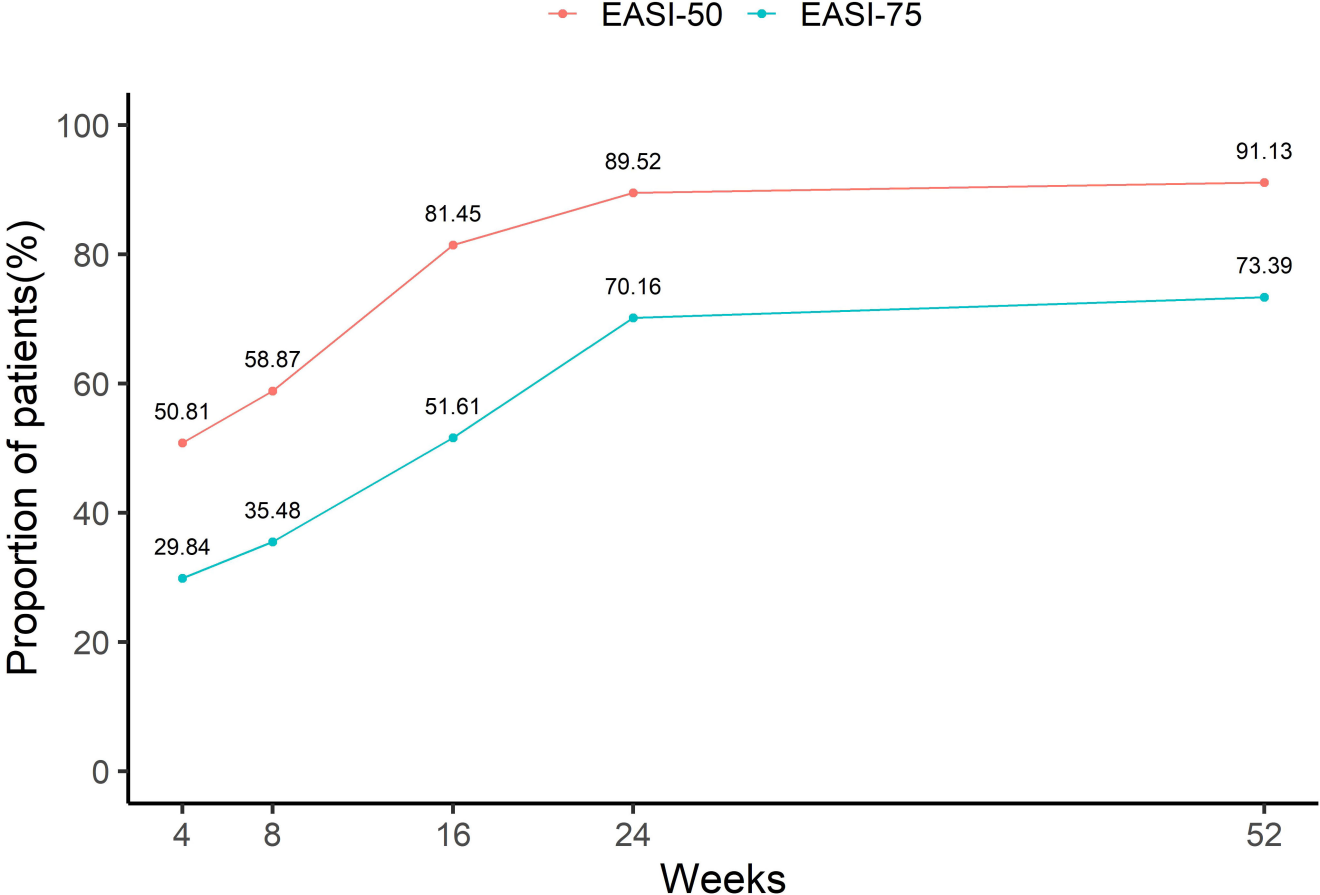
Cumulative proportions of achieving EASI50 and EASI75 within 52 weeks.

**Figure 4** depicts the cumulative proportions of patients achieving EASI 50 and EASI 75 at different time points. During the first 16 weeks, the cumulative proportion of patients achieving EASI 50 and EASI 75 increased rapidly, reaching 81.45% and 51.61%, respectively. Although the cumulative proportions of EASI 50 and EASI 75 continued to increase after week 24, the growth rate slowed down. By week 52, 91.13% and 73.39% of patients had cumulatively achieved EASI 50 and EASI 75, respectively.

#### 3.3.4 Predictive factors for long-term efficacy

The predictive factors affecting the long-term efficacy of dupilumab for AD were explored using multivariate logistic regression analysis. As shown in **Table 4**, sex, atopic comorbidities, baseline EASI score, eosinophil increment, and medication compliance were identified as statistically significant predictors for achieving EASI 50 and EASI 75 at week 52. Female patients had higher odds of achieving EASI 50 (OR: 4.96, 95% CI: 1.47, 19.2, p=0.014) and EASI 75 (OR: 3.77, 95% CI: 1.36, 11.4, p=0.013) compared to male patients. The presence of atopic comorbidities was associated with lower odds of achieving EASI 50 (OR: 0.23, 95% CI: 0.06, 0.79, p=0.025) and EASI 75 (OR: 0.25, 95% CI: 0.08, 0.68, p=0.010).

**Table 4:** Multivariate logistic regression analysis of predictive factors for achieving EASI 50 and EASI 75 efficacy at Week 52.

			Multivariable Logistic Regression for EASI 50		Multivariable Logistic Regression for EASI 75	
Characteristic	N	Event N	OR(95% CI)	p-value	OR(95% CI)	p-value
Sex	121	52				
Male	81	31	—		—	
Female	40	21	4.96 (1.47, 19.2)	0.014	3.77 (1.36, 11.4)	0.013
Age groups	121	84				
<18 years old	18	13	—		—	
18-64 years old	72	49	0.64 (0.12, 3.21)	0.598	0.87 (0.21, 3.83)	0.846
≥65 years old	31	22	0.27 (0.03, 1.76)	0.183	0.52 (0.10, 2.62)	0.425
Overweight BMI	121	84				
No	86	58	—		—	
Yes	35	26	0.97 (0.29, 3.26)	0.963	1.08 (0.39, 2.99)	0.879
Disease duration	121	84				
<72 months	57	44	—		—	
≥72 months	64	40	0.38 (0.13, 1.07)	0.073	1.03 (0.42, 2.54)	0.957
Atopic comorbidities	121	84				
No	64	46	—		—	
Yes	57	38	0.23 (0.06, 0.79)	0.025	0.25 (0.08, 0.68)	0.010
History of immunosuppressant use	121	84				
No	79	53	—		—	
Yes	42	31	1.52 (0.50, 4.80)	0.464	1.76 (0.68, 4.64)	0.243
Baseline EASI score	121	84	1.31 (1.10, 1.61)	0.006	1.16 (1.04, 1.33)	0.017
Baseline SCORAD score	121	84	0.96 (0.89, 1.03)	0.280	0.96 (0.91, 1.02)	0.251
Response at Week 4	121	84				
No	7	3	—		—	
Yes	114	81	0.95 (0.10, 10.4)	0.963	0.74 (0.09, 9.23)	0.793
Eosinophil increment	121	84				
No	47	37	—		—	
Yes	30	18	0.13 (0.03, 0.52)	0.006	0.55 (0.16, 1.74)	0.313
Medication compliance	121	84				
Compliant throughout 52 weeks	87	53	—		—	
Non-compliant in first 16 weeks/Non-compliant from week 17-52			0.10 (0.02, 0.44)	0.006	0.20 (0.06, 0.57)	0.004
Non-compliant throughout 52 weeks		31	0.04 (0.00, 0.25)	0.002	0.08 (0.02, 0.30)	<0.001

OR, Odds Ratio; BMI,Body Mass Index; EASI,Eczema Area and Severity Index; SCORAD, SCORing Atopic Dermatitis.

Additionally, patients with higher baseline EASI scores were more likely to achieve EASI 50 (OR: 1.31, 95% CI: 1.10, 1.61) and EASI 75 (OR: 1.16, 95% CI: 1.04, 1.33) than those with lower baseline EASI scores. Patients with eosinophil increment had significantly lower odds of achieving EASI 50 (OR: 0.13, 95% CI: 0.03, 0.52, p=0.006), but its impact on EASI 75 was not statistically significant. Medication compliance was a strong predictor of treatment efficacy. Compared to patients who were compliant throughout the 52-week period, those who were non-compliant in the first 16 weeks or from weeks 17-52 had significantly lower odds of achieving EASI 50 (OR: 0.10, 95% CI: 0.02, 0.44, p=0.006) and EASI 75 (OR: 0.20, 95% CI: 0.06, 0.57, p=0.004). Patients who were non-compliant throughout the 52-week period had even lower odds of achieving EASI 50 (OR: 0.04, 95% CI: 0.00, 0.25, p=0.002) and EASI 75 (OR: 0.08, 95% CI: 0.02, 0.30, p<0.001).

### 3.4 Safety

During the 52 weeks of dupilumab treatment, a total of 19 adverse events occurred in 18 patients (**Table 5**), with an incidence rate of 14.52%. Among these, 17 patients experienced a single adverse event, and one individual experienced two adverse events simultaneously. The most common adverse event was eye discomfort, with a total of 5 related incidents including 3 cases of conjunctivitis, 1 case of cataract, and 1 case of dry eye. The second most frequent event was minor reactions at the injection site, occurring in four individuals. Folliculitis and upper respiratory tract infections were each reported in two patients. Single incidents of adverse events included herpes zoster, fatigue, allergic asthma, esophagitis, gastritis, and urticaria. All adverse events were mild and resolved after symptomatic treatment without necessitating the discontinuation of dupilumab treatment.

**Table 5:** Adverse events occurring during dupilumab treatment.

Adverse events	Value (N = 19)
Injection-site reactions	4
Conjunctivitis	3
Folliculitis	2
Upper respiratory tract infection	2
Herpes zoster	1
Fatigue	1
Allergic asthma	1
Cataract	1
Esophagitis	1
Gastritis	1
Urticaria	1
Eye dryness	1

## 4 Discussion

As the treatment duration of moderate-to-severe AD patients with dupilumab continues to extend, attention to its long-term efficacy and safety has intensified. To our knowledge, our study is the first real-world investigation in China to explore the efficacy and safety of dupilumab over a 52-week period. The results demonstrated that dupilumab treatment leads to sustained and significant improvements in disease severity, symptoms, and quality of life in these patients over 52 weeks.

In terms of efficacy, the proportion of patients achieving EASI 50 and EASI 75 at week 16 was 72.58% and 42.74%, respectively, slightly lower than a previous 16-week study in China which reported a 64.5% EASI 75 response (32).However, the proportions of patients achieving EASI 50 and EASI 75 remained stable from weeks 16-52, with 67.74% and 41.94% at week 52, respectively. These results were lower compared to other long-term studies of dupilumab conducted in Europe (25, 33, 34),which may be attributed to the fact that only 27.42% of patients in our study were compliant with dupilumab treatment. For example, in the long-term registration trial CHRONOS involving patients with moderate-to-severe AD, 73.8% achieved a mean EASI score reduction after 52 weeks of dupilumab treatment (25). Additionally, two long-term studies by Patruno et al. focused on special AD populations—children aged 6-11 years and elderly patients aged 65 years and above (33, 34). The pediatric study showed that 95.6% and 86.8% of patients achieved EASI 50 and EASI 75 at week 52, respectively (34). Meanwhile, the study on elderly patients found an average 87.2% EASI score reduction from baseline after 52 weeks (33). Pruritus is one of the most common and distressing symptoms for AD patients. Data from this study showed that pruritus severity improved during the 52-week dupilumab treatment period, with significant improvements in NRS scores at week 16 that were maintained at lower levels during weeks 17-52, indicating that dupilumab can quickly and persistently alleviate pruritus symptoms in AD patients. Similarly, patients’ quality of life also significantly improved during the 52-week treatment period.

This study identified several predictive factors associated with the long-term efficacy of dupilumab treatment in moderate-to-severe AD, as measured by the achievement of EASI 50 and EASI 75 at 52 weeks. Female patients demonstrated significantly higher odds of achieving both EASI 50 and EASI 75 compared to male patients. This gender difference in treatment response is consistent with observations from previous studies on dupilumab for AD (32, 35). The presence of atopic comorbidities was associated with lower odds of achieving EASI 50 and EASI 75 in our study. This finding aligns with prior research suggesting that AD patients with comorbid atopic conditions, such as asthma and allergic rhinitis, may exhibit more severe and treatment-resistant disease (36). Our results underscore the importance of assessing and managing atopic comorbidities in AD patients receiving dupilumab. Furthermore, patients with higher baseline EASI scores were more likely to achieve EASI 50 and EASI 75, indicating that dupilumab demonstrates good efficacy for severe AD patients. However, it is important to note that patients with severe AD may still have significant residual disease activity despite achieving these relative improvement thresholds.

Our study also found that elevated eosinophil counts were associated with a decreased likelihood of achieving EASI 50. Previous research utilizing blood transcriptome analysis has identified two endotypes among moderate-to-severe atopic dermatitis patients: eosinophil-high and eosinophil-low (37). The eosinophil-high endotype exhibited significantly elevated expression of genes related to eosinophil signaling pathways in peripheral blood, such as IL5RA, CCL23, and IL34 (37). This endotype also displayed more extensive transcriptome dysregulation compared to the eosinophil-low endotype, which showed less pronounced transcriptomic changes. The proportion of super-responders was slightly lower in the eosinophil-high endotype (37).

Medication compliance emerged as a strong predictor of long-term dupilumab efficacy in our study. Patients who were non-compliant, either in the first 16 weeks or throughout the 52-week period, had significantly lower odds of achieving EASI 50 and EASI 75 compared to compliant patients. This finding underscores the critical importance of adherence to dupilumab dosing schedules for optimal long-term disease control. Although some studies suggested that appropriately extending the treatment interval of dupilumab could still achieve satisfactory efficacy (38, 39), we recommend that patients adhere to the regular treatment regimen within 52 weeks to improve efficacy.

In terms of safety, dupilumab demonstrated a favorable risk profile over the 52-week period, with an overall adverse event rate of 14.52%, comparable to other real-world studies (33, 40). Most events were mild and did not require treatment discontinuation. The most common adverse events were eye discomfort and injection site reactions, which aligns with the known safety profile of dupilumab (33, 40).

One of the key strengths of our study is its prospective, real-world design with an extended 52-week follow-up duration. This addresses a current gap in evidence regarding the long-term use of dupilumab in Chinese clinical practice. However, our study also has some limitations that should be acknowledged. Firstly, all patients were recruited from a single center, and the results may not be generalizable to different regions. Secondly, we only included patients who used dupilumab for up to 12 months, and a large number of patients who used dupilumab for less than 12 months were excluded. This may introduce selection bias.Additionally, China’s medical insurance policy for the indication of dupilumab targets moderate-to-severe AD patients, defined as having a SCORAD score ≥25. Our study included some patients who may have had milder skin lesions (EASI<8) but severe pruritus, resulting in a SCORAD score greater than 25. This should be taken into account when interpreting the results. Finally, 73.39% of patients in our study concurrently used topical medications such as corticosteroids. While reflecting real-world practice, this may have enhanced the treatment efficacy observed in these patients.

## 5 Conclusion

In conclusion, our study provides compelling real-world evidence supporting the durable efficacy and favorable safety profile of dupilumab over a 52-week period for Chinese patients with moderate-to-severe AD. Furthermore, we identified important predictive factors that can guide treatment optimization strategies to enhance long-term therapeutic outcomes.

## Data Availability

The raw data supporting the conclusions of this article will be made available by the authors, without undue reservation.
